# Photodynamic therapy and tumor imaging of hypericin-treated squamous cell carcinoma

**DOI:** 10.1186/1477-7819-4-87

**Published:** 2006-12-05

**Authors:** Christian S Head, Quang Luu, Joel Sercarz, Romaine Saxton

**Affiliations:** 1 Division of Head & Neck Surgery, David Geffen School of Medicine at UCLA, Los Angeles, California 90095, USA

## Abstract

**Background:**

Conventional cancer therapy including surgery, radiation, and chemotherapy often are physically debilitating and largely ineffective in previously treated patients with recurrent head and neck squamous cell carcinoma (SCC). A natural photochemical, hypericin, could be a less invasive method for laser photodynamic therapy (PDT) of these recurrent head and neck malignancies. Hypericin has powerful photo-oxidizing ability, tumor localization properties, and fluorescent imaging capabilities as well as minimal dark toxicity. The current study defined hypericin PDT *in vitro *with human SCC cells before the cells were grown as tumor transplants in nude mice and tested as a model for hypericin induced tumor fluorescence and PDT via laser fiberoptics.

**Methods:**

SNU squamous carcinoma cells were grown in tissue culture, detached from monolayers with trypsin, and incubated with 0.1 μg to 10 μg/ml of hypericin before exposure to laser light at 514, 550, or 593 nm to define optimal dose, time, and wavelength for PDT of tumor cells. The SCC cells also were injected subcutaneously in nude mice and grown for 6–8 weeks to form tumors before hypericin injection and insertion of fiberoptics from a KTP532 surgical laser to assess the feasibility of this operating room instrument in stimulating fluorescence and PDT of tumors.

**Results:**

*In vitro *testing revealed a hypericin dose of 0.2–0.5 μg/ml was needed for PDT of the SCC cells with an optimal tumoricidal response seen at the 593 nm light absorption maximum. *In vivo *tumor retention of injected hypericin was seen for 7 to10 days using KTP532 laser induced fluorescence and biweekly PDT via laser fiberoptics led to regression of SCC tumor transplants under 0.4 cm^2 ^diameter, but resulted in progression of larger size tumors in the nude mice.

**Conclusion:**

In this preclinical study, hypericin was tested for 514–593 nm dye laser PDT of human SCC cells *in vitro *and for KTP532 surgical laser targeting of SCC tumors in mice. The results suggest hypericin is a potent tumor imaging agent using this surgical laser that may prove useful in defining tumor margins and possibly in sterilizing post-resection margins. Deeply penetrating pulsed infrared laser emissions will be needed for PDT of larger and more inaccessible tumors.

## Background

Current estimates indicate that 30,000 people will be diagnosed with squamous cell carcinoma (SCC) of the head and neck region each year in the USA and greater than 8,000 of these patients will die of their disease. Despite recent advances in surgical reconstruction permitting larger resections in addition to new radiation and chemotherapy regimes, the morbidity and mortality of SCC has not decreased significantly in the last 20 years. The median age of death remains 68 years of age and over 50% of head and neck SCC patients recur within 5 years of treatment. For recurrent SCC, the five-year survival rate is less than 10% [1]. Conventional cancer therapies including surgery, radiation, and chemotherapy often are physically debilitating and largely ineffective in previously treated recurrent head and neck tumors. Recent use of laser thermal therapy to palliate these unresectable cancers has not proved effective in reducing the morbidity and mortality of recurrent head and neck cancer patients.

The natural photosensitizing agent, hypericin, may provide a novel approach for less invasive treatment of locally recurrent head and neck tumors [2-4]. Hypericin has powerful photo-oxidizing ability, tumor-seeking characteristics, and minimal dark toxicity, so it is potentially an ideal therapeutic agent for treatment of recurrent SCC. Previous preclinical studies have shown that use of hypericin in photodynamic therapy (PDT) for cancer treatment is effective and may prove to be a clinically valuable procedure in targeting unresectable tumors via this minimally invasive therapy [2-6]. Recently, we found hypericin PDT of cultured human SCC cells *in vitro *can be activated by visible laser light or infrared pulsed laser excitation [7]. In the current study a low power variable wavelength dye laser was used for hypericin-induced PDT of human SCC cells *in vitro *and a KTP532 laser tested for effects on SCC cells grown as tumors in mice.

## Materials and methods

The human squamous carcinoma cell line SNU1 from Seoul National University was grown in tissue culture. SNU cells were cultured in RPMI 1640 medium supplemented with 10% fetal calf serum and 50 ug/ml gentamicin as described previously [3]. Subconfluent monolayers of the cells were detached with 0.25% trypsin in PBS, counted in trypan blue with a hemocytometer, and resuspended in microfuge tubes at 500,000 cells/0.5 ml in RPMI 1640 media without phenol red. These *in vitro *experiments included hypericin concentrations ranging from 0.1 to 10 μg/mL, dye laser wavelengths of 514, 550, and 593 nm, laser power at the fiberoptic tip of 50, 100, and 150 mW with illumination times of 0–120 seconds for a total light fluence of 0–60 J/cm^2 ^measured at the sample tube liquid interface. After PDT each sample was added to microplate wells at 50,000 cells/0.2 ml in triplicate and incubated for 48 hours at 37°C before cell viability was measured by adding 0.5 mg/ml MTT tetrazolium bromide for 3 hours followed by media aspiration, addition of dimethyl sulfoxide (DMSO) to solubilize the dye product, and optical density of each well recorded at 450 nm with a Molecular Devices microplate reader [3].

**Figure 1:**
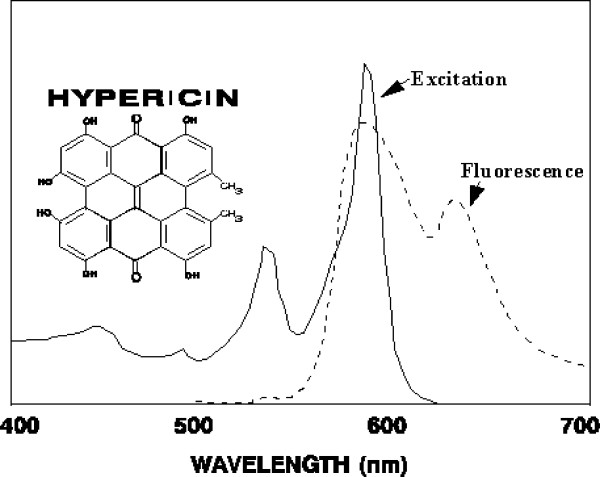
Visible light absorbance and fluorescence spectrum of hypericin in DMSO.

*In vivo *experiments were performed on 3–15 mm diameter tumors growing in athymic nude mice 6–8 weeks after subcutaneous injection of 10^6 ^human SCC cells. Tumors were injected with 10 μL of DMSO containing 10 μg hypericin per gm tumor and after 24 hours treated via insertion of a fiberoptic into the tumor center to deliver KTP 532 nm laser light at 500 mW output from a Laserscope operating room medical laser. KTP safety goggles allowed detection of the orange-red dye tumor fluorescence. Hypericin spectra in DMSO had absorbance maxima at 545 and 593 nm and a red-orange fluorescence emission maxima at 594 and 640 nm as shown in Figure 1.

## Results

This preclinical evaluation of hypericin was carried out to assess its potential use as a new agent for sterilizing postoperative resection margins in adjuvant treatment of recurrent head and neck SCC where specific tumor targeting by photo-activation is needed to avoid normal tissue damage to adjacent vital structures. Hypericin was tested in the cultured SCC cells to define drug and light dosage as well as light wavelength, exposure time, and laser power delivery needed for tumoricidal effects. Both the *in vitro *and *in vivo *experiments described in this study shows that the photosensitizing effects of hypericin induced significant killing of human SCC when excited by visible light from a laser delivered via fiberoptics.

**Figure 2:**
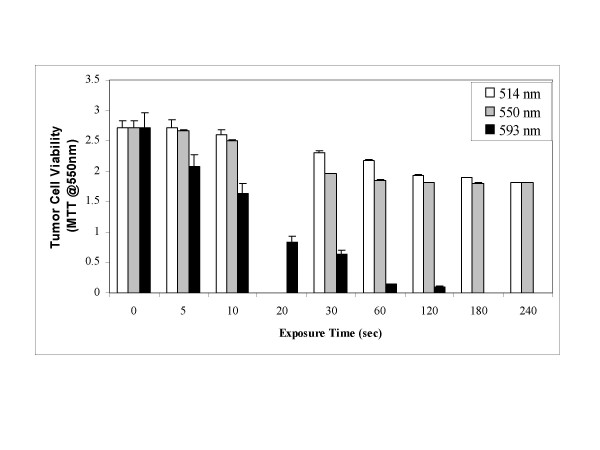
Hypericin phototoxicity in SCC cells at 3 dye laser light emission wavelengths.

Hypericin singlet oxygen mediated phototoxicity in the SCC cells was tested initially with the dye laser at a low power of 50 mW using 3 wavelengths including green 514 nm, yellow 550 nm, and orange 593 nm light. As shown in Figure 2, both 514 nm and 550 nm low intensity laser light induced increased SCC cell death after prolonged illumination, but 593 nm light was tumoricidal at exposure times of 5–10 seconds with nearly complete cancer cell killing after 120 seconds.

**Figure 3:**
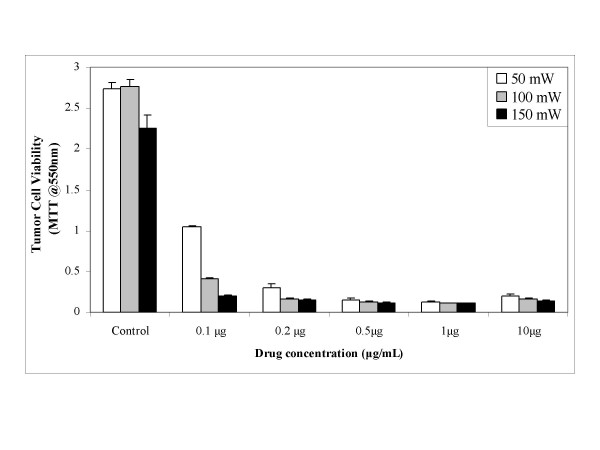
Hypericin dose and laser light fluence effects on SCC cell phototoxicity.

Drug phototoxicity in SCC cells then was tested at varying hypericin concentrations and 593 nm laser light to assess dose response. As shown in Figure 3, decreased SCC cell viability was detectable even at the lowest hypericin dose of 0.1 μg/mL and intense phototoxicity was observed at 0.2 μg/mL, compared to light exposed cells without drug. At a constant light exposure time of 60 seconds and the minimal 0.1 μg/mL drug level, cytotoxicity increased with laser power delivery. These *in vitro *results show a dose-response relationship exists in hypericin sensitized SCC cells with elevated phototoxicity seen as either the drug or light dosages were increased.

**Figure 4:**
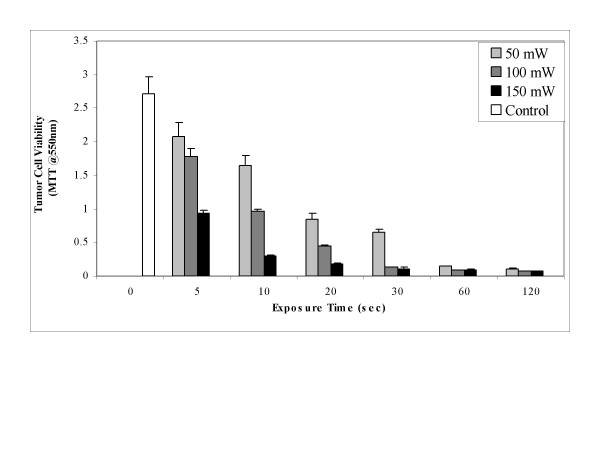
Hypericin PDT of SCC cells at increasing exposure times and laser power.

Next, photoactivation energy was varied after SCC cells were sensitized with only 0.1 μg/mL hypericin and tumoricidal responses to 593 nm light tested by increasing irradiation times and laser power delivery from 50 to 100 and 150 mW. The results in Figure 4 show significant tumor cell killing at 150 mW after only 5–20 seconds light exposure (0.75–3.0 Joules). The 50 mW and 100 mW laser power level induced drug phototoxicity following longer light exposure times.

**Figure 5:**
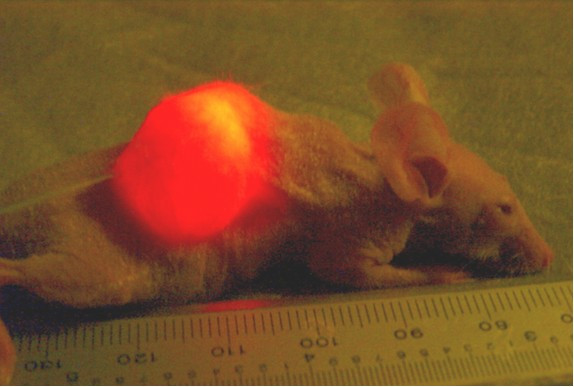
Hypericin in SCC tumor of nude mouse viewed by laser-induced fluorescence.

**Figure 6:**
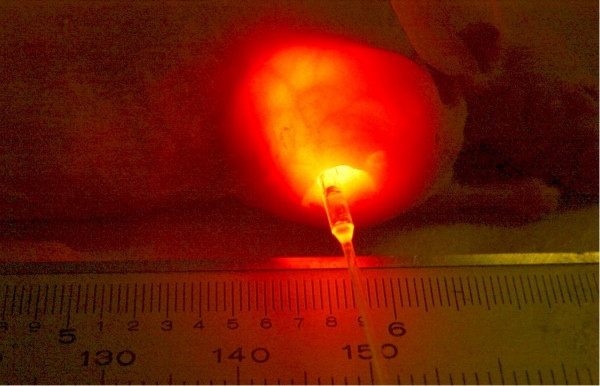
Hypericin intratumor fluorescence induced by laser fiberoptic cylindrical tip.

After identifying optimal dosages for hypericin sensitization and phototoxicity of SCC cells *in vitro*, hypericin was then tested for uptake by the human SCC transplants grown for 4–8 weeks as subcutaneous tumors in mice. Hypericin injected into these subcutaneous tumors diffused rapidly to the margins and remained for up to 10 days when monitored by laser-induced fluorescence as shown below in Figure 5. Insertion of a bare fiberoptic allowed delivery of KTP532 laser green light into the tumor site inducing intense red-orange fluorescence of hypericin when viewed with KTP532 safety goggles. Enhanced hypericin fluorescence also was observed in the tumor by inserting a cylindrical fiberoptic tip redirecting the 532 nm laser photons at right angles from the fiber axis as shown in Figure 6. These results confirmed that hypericin dye diffuses into and is retained by these SCC tumors for prolonged periods. The commonly available KTP532 medical laser also was highly effective in exciting hypericin fluorescence and appears likely to be useful in defining tumor margins during resection of head and neck cancer in the operating room.

**Figure 7:**
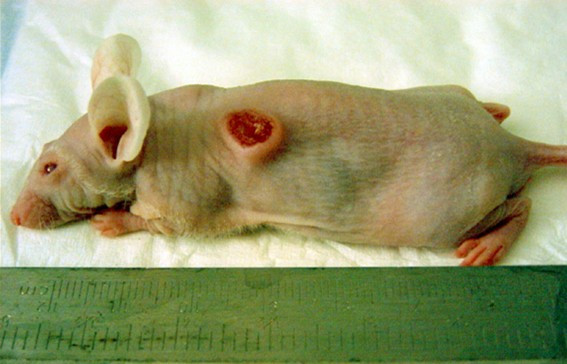
Open SCC tumor in nude mouse prior to hypericin and KTP laser treatment.

**Figure 8:**
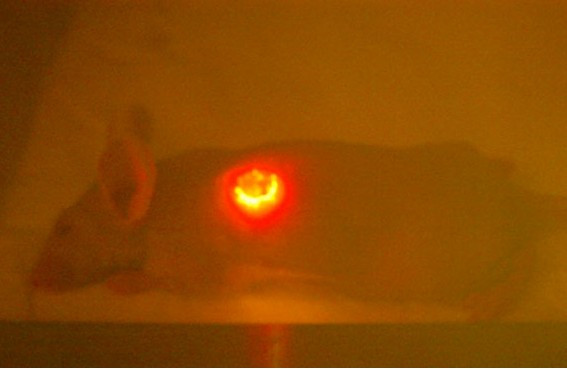
Tumor viewed via KTP532 safety goggles during hypericin and laser phototherapy.

**Figure 9:**
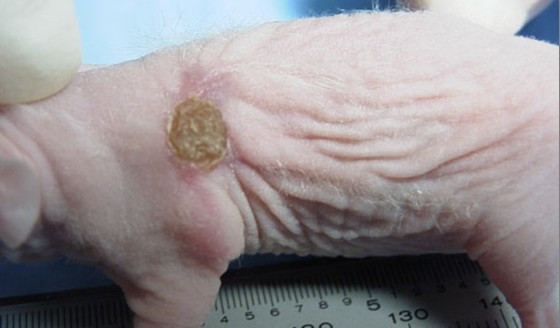
Tumor two months after semi-weekly cycles of hypericin and KTP laser PDT.

Direct *in vivo *laser PDT also was tested on mice after weekly intratumor injection of hypericin. Of 30 SCC tumors evaluated by repeated PDT over 6–8 weeks via KTP532 laser fiberoptic insertion, we observed gradual tumor ablation in half of the treated mice. Use of the fiberoptic diffuser tip in phototherapy of several SCC tumors was not an improvement. In one case an open tumor was injected with hypericin and illuminated biweekly via a bare fiber from above leading to a complete response as shown in Fig 6, 7, 8, 9, 10. Hypericin and laser photo-oxidation proved to be most effective in tumors of smaller size with a volume of 400 mm^3 ^or less, while larger tumors often exhibited partial ablation followed by regrowth. The results show 532 nm light penetration was a limiting factor in PDT of larger tumors and suggest hypericin may be most effective in defining tumor margins by laser induced fluorescence and sterilizing the post-resection field.

**Figure 10:**
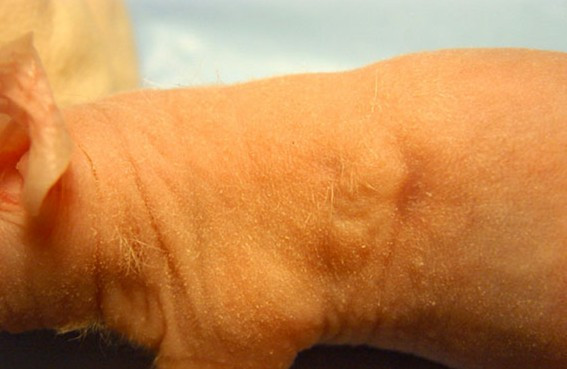
Tumor regression three months after hypericin and KTP laser PDT treatment.

## Discussion

Initial treatment of head and neck squamous cell carcinoma is directed by several considerations with the single most important factor being stage of disease [1]. However, with the advent of newer chemoradiation protocols, an increase in the number of recurrent squamous cell carcinomas has been seen. These recurrent patients usually have no surgical options and need newer modalities of treatment in hopes of palliation after receiving maximum dose radiation or appearance of drug resistant tumors [1]. Tumor imaging and PDT with hypericin have been tested *in vivo *for therapy of pancreas and skin cancer as well as in nasopharyngeal carcinomas [5,8,11]. Experimental studies have shown that hypericin photodynamic therapy is tumoricidal for human cancer cells both *in vitro *and *in vivo *with potential application in targeting recurrent or unresectable malignancies through this minimally invasive new adjuvant procedure [2-7].

The current study supports previously reported data and provides new evidence that hypericin may prove to be clinically useful for both tumor imaging and in PDT of locally recurrent head and neck squamous cell carcinoma. As seen in Figs 5 and 6, hypericin is effective as a fluorescent tumor imaging agent when activated by a conventional operating room KTP532 surgical laser. In an earlier study, we reported that intravenous hypericin injection resulted in significant uptake by SCC tumors, but higher levels of this fluorescent dye were deposited in the lungs and other vascular organs [2]. Although 532 nm laser light was sufficient for hypericin excitation and in fluorescent tumor imaging, the *in vitro *SCC experiments of the current study show clearly that the higher 593 nm laser wavelength is superior in phototherapy. This conclusion also was indicated by a decreased PDT response observed for large SCC tumors after hypericin and KTP532 laser treatment even when fiberoptics were inserted directly into the tumor center to maximize the light delivery. Recent reports show hypericin phototherapy can be enhanced by multi-fraction treatment, by the addition of synthetic oxygen carriers, or by co-administration of a cyclo-oxygenase inhibitor to block prostaglandin synthesis and prevent new angiogenesis in tumor sites [9-11]. Further improvement of hypericin PDT also may be possible using 2-photon infrared pulsed laser emissions to increase tissue penetration of light during tumor imaging and treatment [7]. These advances will make hypericin and lasers a more effective cancer therapy.

## Conclusion

The photodynamic response of hypericin dye was tested *in vitro *with cultured human squamous carcinoma cells after exposure to laser emissions at green, yellow, and orange visible light wavelengths (514, 550, and 593 nm). This study revealed that the greatest PDT responses occurred at hypericin's absorption and fluorescence maximum of 593 nm. A range of hypericin concentrations was tested to determine the minimum dosage for photosensitization of the tumor cells, which was found to be from 0.2 to 0.5 μg/mL or 1 μM. Laser power delivery at 150 mW produced the highest level of tumor cell killing at 593 nm. *In vivo *studies with nude mice bearing human squamous cell carcinoma tumors showed that injected hypericin remained within the tumor site for 10 days allowing repeated laser illumination.

This study also confirms that placement of the fiberoptic within the tumor provides the greatest photo-oxidation and is the optimal route of laser light delivery. During these PDT tests of subcutaneous human SCC tumors using a surgical KTP532 laser emitting visible green light, the limiting factor appears to be tumor size. Hypericin PDT also should be enhanced via deeper penetration of 593 nm light as in our *in vitro *studies above or by using longer wavelengths of pulsed infrared light for 2-photon excitation [7]. This study provides new evidence hypericin is an ideal photosensitizer for PDT that may prove to be clinically useful in tumor imaging and minimally invasive treatment of locally recurrent head and neck squamous cell carcinoma.

## Competing interests

The author(s) declare that they have no competing interests.

## Authors' contributions

CH conceived of the study, and participated in its design and coordination and drafted the manuscript and multiple revisions. He also funded the projected from his grant from NIH. QL participated in the in vitro tumor growth and animal inoculation and treatment and aided in drafting the manuscript. RS and JS participated in design and coordination of this study and drafting the manuscript. RS aided in laser methodology and treatment of animals.

All authors read and approved final manuscript.
